# Hierarchical spin-orbital polarization of a giant Rashba system

**DOI:** 10.1126/sciadv.1500495

**Published:** 2015-09-25

**Authors:** Lewis Bawden, Jonathan M. Riley, Choong H. Kim, Raman Sankar, Eric J. Monkman, Daniel E. Shai, Haofei I. Wei, Edward B. Lochocki, Justin W. Wells, Worawat Meevasana, Timur K. Kim, Moritz Hoesch, Yoshiyuki Ohtsubo, Patrick Le Fèvre, Craig J. Fennie, Kyle M. Shen, Fangcheng Chou, Phil D. C. King

**Affiliations:** 1 SUPA, School of Physics and Astronomy, University of St. Andrews, St. Andrews, Fife KY16 9SS, UK.; 2 Diamond Light Source, Harwell Campus, Didcot OX11 0DE, UK.; 3 School of Applied & Engineering Physics, Cornell University, Ithaca, NY 14853, USA.; 4 Center for Condensed Matter Sciences, National Taiwan University, Taipei 10617, Taiwan.; 5 Institute of Physics, Academia Sinica, Taipei 11529, Taiwan.; 6 Laboratory of Atomic and Solid State Physics, Department of Physics, Cornell University, Ithaca, NY 14853, USA.; 7 Department of Physics, Norwegian University of Science and Technology (NTNU), N-7491 Trondheim, Norway.; 8 School of Physics, Suranaree University of Technology, Nakhon Ratchasima 30000, Thailand.; 9 NANOTEC-SUT Center of Excellence on Advanced Functional Nanomaterials, Suranaree University of Technology, Nakhon Ratchasima 30000, Thailand.; 10 Synchrotron SOLEIL, CNRS-CEA, L’Orme des Merisiers, Saint-Aubin-BP48, 91192 Gif-sur-Yvette, France.; 11 Kavli Institute at Cornell for Nanoscale Science, Ithaca, NY 14853, USA.

**Keywords:** spintronics, Rashba, BiTeI, Spin-orbit coupling, Angle-resolved photoemission, Electronic structure, Orbital texture

## Abstract

The Rashba effect is one of the most striking manifestations of spin-orbit coupling in solids and provides a cornerstone for the burgeoning field of semiconductor spintronics. It is typically assumed to manifest as a momentum-dependent splitting of a single initially spin-degenerate band into two branches with opposite spin polarization. Combining polarization-dependent and resonant angle-resolved photoemission measurements with density functional theory calculations, we show that the two “spin-split” branches of the model giant Rashba system BiTeI additionally develop disparate orbital textures, each of which is coupled to a distinct spin configuration. This necessitates a reinterpretation of spin splitting in Rashba-like systems and opens new possibilities for controlling spin polarization through the orbital sector.

## Teaser

Angle-resolved photoemission reveals the emergence of complex orbital texture concomitant with spin splitting in the Rashba compound BiTeI.

## INTRODUCTION

The ability to generate and control spin splittings of electronic states is a key goal in the search for spintronic materials (*1*). A particularly successful strategy has been the lifting of spin degeneracy via spin-orbit coupling in the presence of structural inversion asymmetry. Termed the Rashba or Bychkov-Rashba effect (*2*), this phenomenon manifests through a spin-momentum locking of the quasiparticles, stabilizing a pair of Fermi surfaces that are typically assumed to exhibit counter-rotating chiral spin textures (*3*). The ability to electrostatically control the strength of such spin splitting (*4*–*7*) has led to prospects for all-electrical manipulation of electron spin precession (*8*), offering new prototypical schemes of semiconductor spintronics (*1*). The quest to practically realize such devices and to operate them without cryogenic cooling has motivated a major search for materials that can host stronger spin splittings than can typically be achieved with conventional semiconductors (*6*, *9*–*12*).

**Fig. 1:**
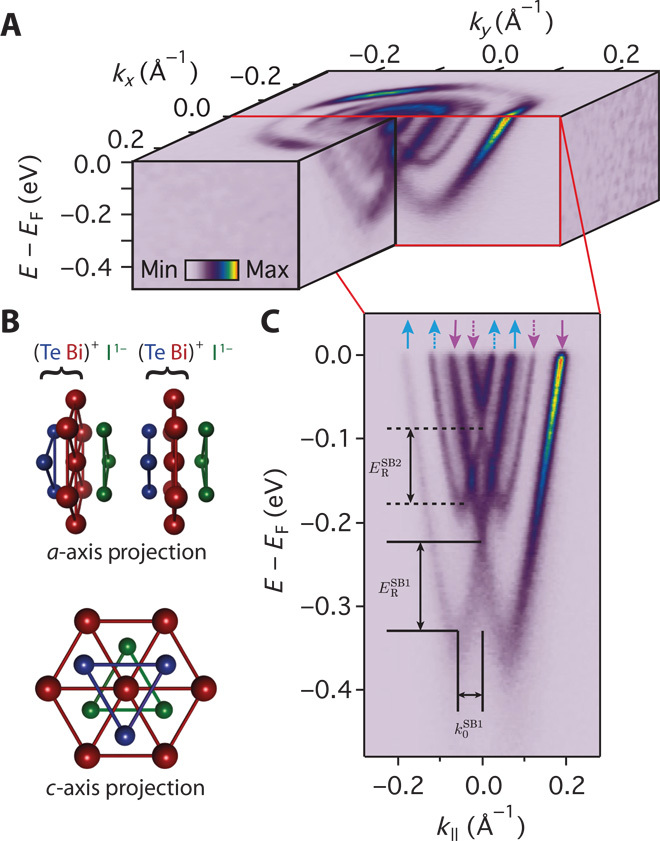
Surface electronic structure of BiTeI. (**A**) ARPES measurements of the Fermi surface and near-*E*_F_ band dispersions measured (*hν* = 52 eV, *p*-polarization) from the Te-terminated surface of BiTeI. (**B**) A lack of inversion symmetry of the bulk crystal structure together with strong spin-orbit coupling mediates a large Rashba-like spin splitting. Additionally, the polar nature of the Te-terminated surface induces a strong downward band bending, causing a ladder of Rashba-split subband states to emerge in the near-surface quantum well. (**C**) These are clearly resolved in measurements of the dispersion along Γ–*M*. The conventional spin texture associated with such Rashba splitting is shown schematically by colored arrows, with the spin expected to lie predominantly in the surface plane.

A giant Rashba-like spin splitting has recently been discovered for bulk conduction and valence band states of bismuth tellurohalide semiconductors (*13*–*15*). Arising because of a combination of bulk inversion asymmetry (Fig. 1B), strong atomic spin-orbit coupling, and a negative crystal field splitting of the valence bands (*14*), Rashba parameters have been uncovered that are among the highest of any known materials, together with a counter-rotating chiral Fermi surface spin texture (*13*, *16*). Exploiting element- and orbital-selective angle-resolved photoemission (ARPES), we show that a complex interplay between atomic, orbital, and spin degrees of freedom significantly enriches this picture. We expect our findings to be broadly applicable across other strong spin-orbit Rashba systems.

## RESULTS

First, we summarize the generic electronic structure of the Te-terminated surface of BiTeI (Fig. 1). Previous measurements have shown this termination to support a near-surface electron accumulation (*13*, *17*). We clearly observe two 2D (two-dimensional) subbands (SB1 and SB2 in Fig. 1C; see also fig. S1) formed within the resulting quantum well. Each subband hosts two branches with a separation that grows approximately linearly with momentum away from their crossing at *k* = 0. This is a hallmark of Rashba-like spin splitting. We extract a large Rashba energy *E*_R_ = 120 ± 10 meV (85 ± 5 meV) and momentum offset of the band bottom *k*_0_ = 0.055 ± 0.005 Å^−1^ (0.050 ± 0.005 Å^−1^) for the first (second) subband, respectively, supporting previous studies that established this material as a model host of giant spin splittings (*13*, *14*, *16*).

**Fig. 2:**
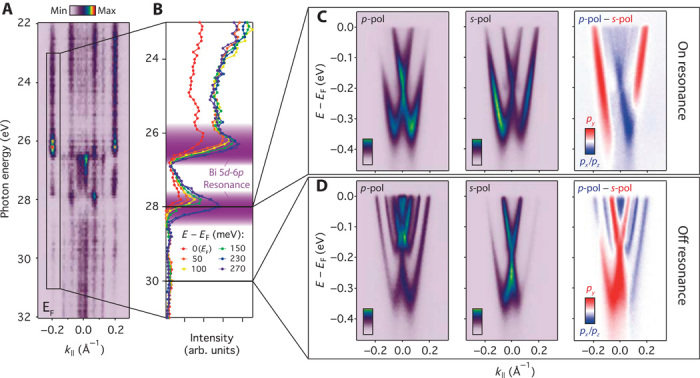
Disentangling intertwined atomic and orbital characters. (**A**) Fermi level momentum distribution curve (*E*_F_ ±15 meV) measured along Γ–*M* as a function of probing photon energy using *p*-polarized light. No dispersion is observed, indicating 2D states consistent with our assignment of quantum well subbands, whereas strong matrix element variations give rise to pronounced intensity modulations. (**B**) Extracted spectral weight of the outermost band (*k*_*F*_ ≈ −0.2 Å^−1^) as a function of binding energy, revealing characteristic intensity enhancement due to resonant photoemission at the Bi O-edge. (**C** and **D**) Corresponding dispersions (along Γ–*M*) measured on-resonance (*hν* = 28 eV) and off-resonance (*hν* = 30 eV), respectively, with *p*-polarized (left) and *s*-polarized (center) light. The difference in spectral weight between these dispersions (right) indicates a band and element-dependent orbital polarization.

Although typically treated in a single-band picture, the electronic wave function for each branch of a Rashba-split state can be more generally written as $\Psi =\sum_{i,\tau ,\sigma }c_{i,\tau }^{\sigma }\psi_{i,\tau }$, where, following the notation in (*18*), *i* is the atomic index, τ ∈ {*p*_*x*_, *p*_*y*_, *p*_*z*_} and σ are the orbital and spin indices, respectively, ψ_*i*,τ_ are atomic wave functions, and $c_{i,\tau }^{\sigma }$ are complex coefficients. Neglecting spin-orbit coupling, our calculations predict a conduction band in BiTeI predominantly derived from Bi *p*_*z*_ orbitals (see fig. S2). Including such effects, however, not only permits it to become strongly spin-split via Rashba-like interactions but also promotes significant orbital mixing. In general, therefore, multiple $c_{i,\tau }^{\sigma }$ can be expected to become nonnegligible. For a complete description of the Rashba-split states, it is therefore essential to consider the interplay of the underlying atomic, orbital, and spin components. To disentangle these contributions, we combine two powerful features of ARPES: characteristic selection rules for photoemission using linearly polarized light, allowing us to directly probe the orbital wave function (*19*, *20*), and resonant photoemission to provide elemental sensitivity (*21*). Such resonant enhancements are evident in Fig. 2 (A and B). They cause the spectral weight of the conduction band states to strongly peak at photon energies around 26 and 28 eV, close in energy to the binding energy of the Bi 5*d*_5/2,3/2_ core levels, with functional forms that are well described by Fano line shapes. This points to a significant Bi-derived atomic character of the lowest conduction band states, consistent with theoretical calculations (*22*). We exploit this, selectively probing “on-resonance” to unveil the Bi-projected spectral function.

**Fig. 3:**
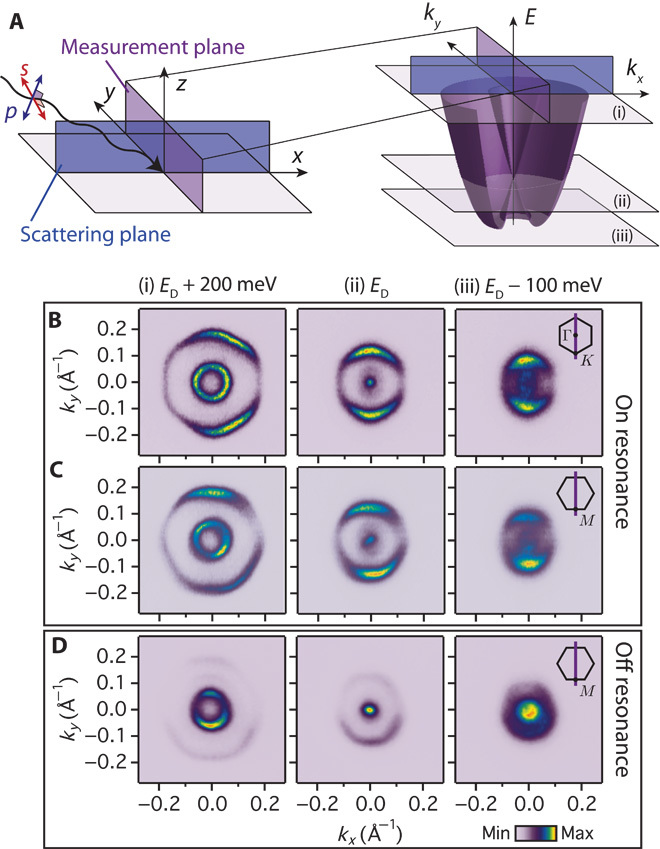
*p*_*y*_-projected spectral weight distribution. (**A**) Experimental geometry for our measurements and (**B** to **D**) resulting CESs measured using *s*-polarized light to probe the *p*_*y*_ orbital character at 200 meV above, exactly at, and at 100 meV below the Dirac point (*E*_D_) formed by the crossing of the two spin-split branches of the lowest subband. On-resonance measurements (*hν* = 28 eV) with the scattering plane aligned to (B) Γ–*M* and (C) Γ–*K*, and (D) off-resonance (*hν* = 30 eV, scattering plane along Γ–*K*) measurements show pronounced angular variations in spectral weight, characteristic of strongly momentum-dependent orbital textures.

The resulting measurements of the dispersion reveal pronounced momentum-dependent spectral weight variations (Fig. 2C). We focus on the first subband (SB1), which is most clearly visible across our measurements. Measurements using *p*-polarized light yield stronger spectral weight for the inner branch of this subband, whereas the outer branch is significantly more pronounced when probed using *s*-polarization. Selection rules (*19*) dictate that, of the *p* orbitals, the former measurement should be sensitive to *p*_*z*_- and *p*_*x*_-derived orbital character, whereas the transition matrix element is only nonvanishing for photoemission from *p*_*y*_ orbitals in the latter case (our measurement geometry is shown in Fig. 3A). The asymmetric spectral weight distributions within and between these measurements immediately establish that the two spin-split branches of the dispersion host a markedly different orbital makeup. Moreover, when measuring with a photon energy only 2 eV higher (Fig. 2D), we find an almost complete reversal of these matrix element variations, with greater spectral weight for the inner branch of the lowest subband when measuring using *s*-polarized light. No longer on resonance, these measurements will not a priori be dominated by the Bi-derived spectral weight, and we therefore conclude that the orbital textures of the Rashba-split states are also strongly dependent on their underlying atomic character, as discussed in detail below.

Figure 3 (B and C) shows constant binding energy surfaces (CESs) measured on-resonance with *s*-polarized light to selectively probe the momentum-space distribution of the Bi *p*_*y*_ orbital character. The outer band of each CES exhibits strong spectral weight along our measurement direction (at positive and negative *k*_*y*_), with a pronounced suppression at positive and negative *k*_*x*_, over the entire occupied bandwidth of the conduction bands. This remains qualitatively unchanged under rotation of the azimuthal orientation of the sample. Such behavior is indicative of a *p*_*y*_-like orbital aligned along our measurement direction, irrespective of whether the Γ–*M* or Γ–*K* crystallographic direction is oriented to this (*18*, *20*). The in-plane Bi-projected charge density must therefore be oriented such that its lobes predominantly point radially outward from the outer CES.

**Fig. 4:**
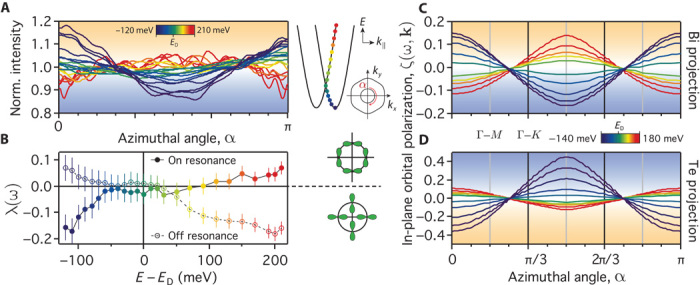
Mapping the angle-dependent orbital wave functions. (**A**) Angular distribution of the Bi *p*_*y*_-projected spectral weight distribution measured on-resonance around the inner band of CESs (see, for example, Fig. 3B), each normalized to its average value. Above (below) the Dirac point, *E*_D_, this is peaked at an azimuthal angle of α = π/2 (0 and π) indicative of a tangential (radial) in-plane orbital alignment. (**B**) The smooth evolution between these two configurations is captured by the relative spectral weight ADF, λ(ω) = [*I*_α=π/2_(ω) − *I*_α=0,π_(ω)]/[*I*_α=π/2_(ω) + *I*_α=0,π_(ω)], which crosses zero at the Dirac point within our experimental error. (**C**) This behavior is fully captured by the momentum-dependent Bi *p*_*y*_:*p*_*x*_ in-plane orbital polarization, ζ(ω, **k**), extracted from our density functional theory (DFT) calculations. (**D**) The in-plane orbital polarization is reversed for the Te-projected component, with radial alignment above the Dirac point and tangential alignment below *E*_D_, as also captured by the opposite ADF measured off-resonance (*hν* = 30 eV; see Fig. 3C, summarized in B).

A similar conclusion can be drawn for the inner band of the CES at energies below the Dirac point formed by the crossing of the two spin-split branches of the dispersion. As already evident 100 meV below the Dirac point in Fig. 3 (B and C), and more clearly shown in the angular dependence of spectral weight extracted around the CES in Fig. 4A, the spectral weight still peaks along the *k*_*y*_ direction (α = 0 and π in Fig. 4A), with a suppression of spectral weight along *k*_*x*_ (α = π/2). Indeed, whereas the band edge turning point in the dispersion leads to a van Hove singularity in the density of states (*23*), the inner band of the CES below the Dirac point smoothly evolves into the outer band as it moves through this turning point, and thus hosts qualitatively the same orbital texture.

However, moving up through the Dirac point, the angular distribution of spectral weight around the inner CES gradually flattens and then inverts to become peaked at α = π/2. This points to a tangential alignment of the in-plane Bi-derived orbital character above the Dirac point, with stronger *p*_*y*_ character at positive and negative *k*_*x*_ than at positive and negative *k*_*y*_. This behavior is well reproduced by our first-principles calculations. As shown in Fig. 4C, the in-plane *p*_*y*_:*p*_*x*_ orbital polarization around the calculated CESs of the lower subband shows a clear peak at α = π/2 above the Dirac point, and 0 and π below. To capture this generic behavior, we extract the relative spectral weight angular distribution factor (ADF) from our measurements:

$$\lambda (\omega )=\frac{I_{\alpha =\pi /2}(\omega )-I_{\alpha =0,\pi }(\omega )}{I_{\alpha =\pi /2}(\omega )+I_{\alpha =0,\pi }(\omega )},$$ (1)

where *I*_α_(ω) is the spectral weight at angle α and energy ω. This provides an experimental measure of the calculated “orbital polarization ratio” used to describe topological states in Cao *et al.* (*20*). Here, this parameterizes the relative strength of spectral weight, and thus *p*_*y*_ orbital character, along the *k*_*x*_ and *k*_*y*_ directions of our measured CESs, reflecting the strength (|λ|) and alignment of radial (λ < 0) and tangential (λ > 0) in-plane orbital textures. As shown in Fig. 4B, we find that λ(ω) is positive at energies above the Dirac point for the on-resonance measurements, consistent with the dominantly tangential in-plane Bi-projected charge density distribution of the inner band assigned above. With decreasing energy toward the Dirac point, λ is suppressed to zero, indicating a loss of orbital polarization, before growing again but with opposite sign below the Dirac point. Thus, we have experimentally observed a gradual crossover from a radial to a tangential alignment of the in-plane Bi orbital character with the switch occurring exactly at the Dirac point within our experimental error. This is strikingly similar to a recently reported switch in orbital texture at the Dirac point of the spin-polarized surface states of topological insulators (*18*, *20*). Our observation of such a crossover in a topologically trivial compound establishes this as a general feature of strongly spin-orbit coupled systems, a point we return to below.

Intriguingly, extracting λ(ω) from our measurements performed off-resonance, we find an opposite trend (Fig. 4B). Above the Dirac point, λ < 0, indicating a radial alignment of the in-plane orbital-projected charge density, as also clearly evident in the spectral weight distribution visible in Fig. 3D. This is again smoothly suppressed to zero approaching the Dirac point before becoming positive, revealing a tangential orbital configuration, at energies below the Dirac point. We attribute this as a signature of the in-plane Te orbital polarization, which matches well with that found in our calculations (Fig. 4D). At 30 eV photon energy, there is no Bi resonant enhancement and the photoemission cross section is higher for Te *5p* than Bi *6p* orbitals (*24*). Moreover, Te is located right at the surface, and so there is no depth dependent attenuation of Te-derived spectral weight due to the surface sensitivity of photoemission. Our calculations (fig. S2) reveal a strong in-plane Te character of the inner branch of the dispersion, which can thus dominate the spectral weight of the inner branch of the CES in these off-resonance measurements. However, for the outer branch, there is little calculated in-plane Te weight (fig. S2), and so the weak spectral features visible for the outer branch in Fig. 3D are still reflective of the Bi in-plane orbital texture.

## DISCUSSION

**Fig. 5:**
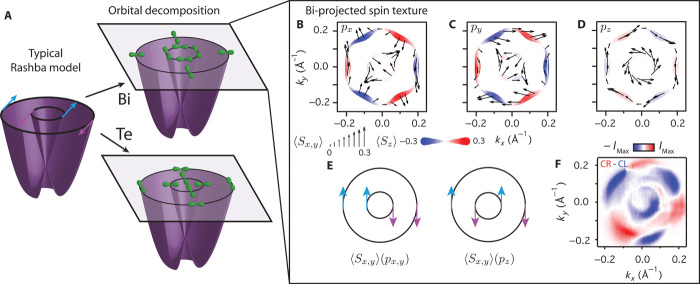
Hierarchy of atomic, spin, and orbital coupling in BiTeI. (**A**) Schematic representation of disparate in-plane orbital textures uncovered for the inner and outer “spin-split” branches of a model Rashba system. (**B** to **D**) Corresponding spin textures calculated from DFT for Bi (B) *p*_*x*_, (C) *p*_*y*_, and (D) *p*_*z*_ projections of CESs 200 meV above the Dirac point. The in-plane spin texture is shown by arrows, and the out-of-plane by the background color, both in units of ℏ/2. (**E**) Schematic of the in-plane spin textures coupled to the net in-plane and out-of-plane orbital textures. (**F**) Circular dichroism measurements performed on-resonance (*hν* = 28 eV, CES at *E*_D_ + 200 meV), revealing a significant sixfold modulation for the outer band indicative of pronounced out-of-plane spin canting.

Together, these measurements and calculations reveal that spin-orbit coupling induces a complex atomic and momentum-dependent hierarchy of orbitally polarized components of the underlying electronic structure in BiTeI, summarized schematically in Fig. 5. Our calculations additionally reveal how each orbital component is, in turn, coupled to a disparate spin texture. We illustrate this for the Bi-derived states in Fig. 5 (B to D); the Te-derived component is additionally shown in fig. S2. The in-plane spin-texture 〈*S*_*x*,*y*_〉 projected onto Bi *p*_*z*_ orbitals yields a conventional counter-rotating chiral spin texture of neighboring CESs at energies above the Dirac point, characteristic of classic Rashba systems (*2*, *3*) and indeed experimentally observed for BiTeI (*13*, *16*). In contrast, the spin texture is significantly more complex for the *p*_*x*_ and *p*_*y*_ orbital projection, with the in-plane spin component switching between tangential and radial around the CES. This results from a coupling of the spin to the characteristic orbital textures, which is similar to that recently found for topological surface states (*25*–*27*).

For example, combined with our uncovered tangential (radial) in-plane Bi orbital texture above (below) the DP, this leads to a net clockwise spin rotation for the in-plane orbital projection of both the inner and outer CESs. This is a strong departure from the conventional picture of Rashba-like spin polarization. Such in-plane orbital textures likely also underlie the unconventional spin configuration predicted for certain *p*_*x,y*_-derived states in Bi/Cu(111) (*28*) and Pb/Cu(111) (*29*) surface alloys. They are also broadly consistent with recent first-principles calculations that suggest that the Rashba parameter can be orbital-dependent in bismuth tellurohalides (*30*), although here we reveal how orbital mixing can lock the spin components on the nominally “spin-split” CESs together, stabilizing pronounced components of the underlying wave function that host markedly non–Rashba-like spin textures in this system. Similar considerations hold for the Te-derived components, although with additional variations in the magnitude of the spin components projected onto out-of-plane versus in-plane orbital components between the CESs due to a greater out-of-plane versus in-plane orbital polarization for Te (figs. S2 and S3). With each orbital component locked to a different spin texture, the fundamental requirement from time-reversal symmetry of spin degeneracy at the Kramer’s point (the Dirac point formed in this system at *k* = 0) ensures equal contribution of in-plane *p*_*x*_ and *p*_*y*_ orbitals at this point. Thus, the vanishing of orbital polarization at the Dirac point experimentally observed above can be simply viewed as an orbital analog of Kramer’s spin degeneracy in time-reversal symmetric systems.

Away from the Dirac point, our calculations additionally predict a strong canting of the spin out of the surface plane for the Bi-derived orbitals. This grows in magnitude with increasing energy away from the Dirac point, where the CESs become increasingly hexagonally warped (*13*). We show evidence for this through circular dichroism measurements. We attribute such dichroism as a signature of unquenched orbital angular momentum (*31*), which our calculations reveal is large and locked approximately opposite to the spin angular momentum because of the strong spin-orbit coupling. Circular dichroism shows a complex dependence on photon energy in this system (*32*) as well as in other layered compounds such as Bi_2_Te_3_ (*33*), which can naturally arise as a consequence of interlayer photoelectron interference (*18*, *27*, *32*). To simplify such effects, we again perform our measurements on-resonance, selectively enhancing states of Bi character (Fig. 5F). We find that the outer (hexagonally warped) band in such measurements develops a pronounced sixfold modulation, which has previously been shown to reflect out-of-plane spin canting in topological insulators (*7*, *34*, *35*). Here, this provides the first experimental evidence for deviations from simple in-plane chiral spin textures in BiTeI.

As evident in Fig. 5 (B to D), 〈*S*_*z*_〉 is substantially larger when projected onto the in-plane orbital components than for the *p*_*z*_ projection. The emergence of a large out-of-plane spin canting in this system is thus intricately tied to the development of the in-plane orbital texture away from the Dirac point observed here. Together, our measurements and calculations establish a powerful role of in-plane atomic orbitals shaping the spin structure of the model Rashba compound BiTeI, revealing a complex interplay between atomic makeup, anisotropic orbital textures, and spin-momentum locking. Whereas small quantitative variations may occur because of near-surface potential contributions to the Rashba spin splitting and possible surface-induced orbital reconstructions, our findings should be broadly applicable to the spin-split bulk electronic structure of BiTeI and should be generic to other strong spin-orbit Rashba systems, suggesting new routes to control spin splitting through the orbital sector. For example, exploiting structure-property relations to tune the competition of atomic spin-orbit coupling and crystal field splitting will allow control over the ratio of in-plane and out-of-plane orbital polarization, thus modulating the degree of non–Rashba-like spin components and out-of-plane spin canting. Together, this promises new prospects for the targeted design of optimized spintronic materials.

## MATERIALS AND METHODS

### Angle-resolved photoemission

ARPES measurements were performed at the CASSIOPEE beamline of SOLEIL synchrotron (France) and I05 beamline of Diamond Light Source (UK). Single-crystal samples of BiTeI, grown by chemical vapor transport, were cleaved in situ at a measurement temperature of 10 K. Measurements were performed using linear-horizontal, linear-vertical, and circularly polarized synchrotron light at the photon energies described in the text and reproduced on multiple samples. Scienta R4000 hemispherical electron analyzers were used, with a vertical entrance slit and the light incident in the horizontal plane, as shown in Fig. 3A. BiTeI has domains of mixed Te and I termination, supporting surface electron and hole accumulation layers, respectively (*17*). By monitoring the relative spectral weight of electron- and hole-like bands crossing the Fermi level and the characteristic core-level shifts in x-ray photoemission spectra, we aligned the synchrotron light spot on Te-terminated domains of the sample. From core-level spectra, we estimate an upper limit of 2% I-terminated regions within our probing region for the data shown here.

### DFT calculations

DFT was performed within the generalized gradient approximation (GGA). We used the DFT code, OpenMX (*36*), based on the linear combination of pseudo-atomic orbitals (LCPAO) method (*37*). Spin-orbit interaction was included via the norm-conserving, fully relativistic *j*-dependent pseudopotential scheme in the noncollinear DFT formalism (*38*). We model the Te-terminated BiTeI surface electronic structure by a supercell calculation including a slab of 60 atomic layers and a vacuum region of ~20 Å thickness. To calculate the spin and orbital angular momentum for specific *k*-points, we used the LCPAO coefficients of local atoms.

## SUPPLEMENTARY MATERIALS

Supplementary material for this article is available at http://advances.sciencemag.org/cgi/content/full/1/8/e1500495/DC1 Fig. S1. Photon energy–dependent ARPES. Fig. S2. Calculated surface electronic structure of BiTeI. Fig. S3. Coupled Te spin-orbital texture. References (*39*, *40*)
